# Local geology controlled the feasibility of vitrifying Iron Age buildings

**DOI:** 10.1038/srep40028

**Published:** 2017-01-12

**Authors:** Fabian B. Wadsworth, Michael J. Heap, David E. Damby, Kai-Uwe Hess, Jens Najorka, Jérémie Vasseur, Dominik Fahrner, Donald B. Dingwell

**Affiliations:** 1 Ludwig-Maximilians-Universität, Munich, Germany; 2 Institut de Physique de Globe de Strasbourg (UMR 7516 CNRS), Strasbourg, France; 3 US Geological Survey, Menlo Park, California, USA; 4 Natural History Museum, London, U.K

## Abstract

During European prehistory, hilltop enclosures made from polydisperse particle-and-block stone walling were exposed to temperatures sufficient to partially melt the constituent stonework, leading to the preservation of glassy walls called ‘vitrified forts’. During vitrification, the granular wall rocks partially melt, sinter viscously and densify, reducing inter-particle porosity. This process is strongly dependent on the solidus temperature, the particle sizes, the temperature-dependence of the viscosity of the evolving liquid phase, as well as the distribution and longevity of heat. Examination of the sintering behaviour of 45 European examples reveals that it is the raw building material that governs the vitrification efficiency. As Iron Age forts were commonly constructed from local stone, we conclude that local geology directly influenced the degree to which buildings were vitrified in the Iron Age. Additionally, we find that vitrification is accompanied by a bulk material strengthening of the aggregates of small sizes, and a partial weakening of larger blocks. We discuss these findings in the context of the debate surrounding the motive of the wall-builders. We conclude that if wall stability by bulk strengthening was the desired effect, then vitrification represents an Iron Age technology that failed to be effective in regions of refractory local geology.

Walled structures, often built from local geological materials^1^,^2^, were a prevalent feature of the European Iron Age. Approximately 200 examples exist which are known to bear evidence for at least partial vitrification^1^,^3^,^4^. The best-studied examples occur in Scotland^1^,^2^. Despite more than a century of academic investigation, the motives for and technology used in this anthropogenic high-temperature process remain a matter of debate. Currently, the leading theories are [1] fire-damage of the enclosure wall sustained during attack or as a result of warfare^5^,^6^, [2] post-occupancy deconstruction, abandonment, or site closure^7^,^8^, [3] a display of prestige or conspicuous consumption^9^, or [4] intentional strengthening of the enclosure by partial melting^1^,^10^,^11^. Here we add experimental constraints to the debate by demonstrating that the rock types available to the builders were a first order control on the feasibility of vitrification, and by confirming that a strengthening effect can indeed ensue after wall burning. The question remains as to whether such strengthening was an intentional constructional process. We thus discuss these findings in the context of the debate on wall vitrification motives.

Previous work has shown that vitrification of Iron Age enclosure walls occurred during prolonged episodes of heating to peak temperatures of 1270–1570 K^4^,^12^,^13^,^14^ for durations >5 hours^13^. Under such conditions, most of the rock types from which such prehistoric walls were built undergo partial or complete melting^12^, and have yielded upon cooling, a glass, or the devitrified products thereof. The temperature at which equilibrium partial melting initiates –the solidus– is highly dependent on the bulk composition and mineralogy of the enclosure wall rocks^15^. By constraining the temperatures and timescales of vitrification, for a wide range of fort-wall compositions, we attempt here to replicate the thermal process that led to fort vitrification in antiquity and to examine its consequences for both the thoroughness of vitrification and the bulk strength of the resultant material.

To achieve this goal we use two archetypes of analogue fort-building materials, [1] a granodiorite (Lanhélin, France) and [2] a sandstone (Darley Dale, U.K.). These materials were selected because of their similarity to lithologies used in a wide range of forts in Europe and because they are already well-studied rock types in rock physics^16^,^17^. Darley Dale—a feldspathic sandstone with subordinate clay—has an average grain size of ~250 μm (Fig. 1a) and a total porosity of ~0.16. Lanhélin granodiorite (comprising predominately quartz, feldspar, and mica) has an average crystal size of ~1000 μm (Fig. 1b) and a total porosity of ~0.01. Below we investigate how the properties of these materials evolve during exposure to high temperatures. We then use simple models to constrain the temperature-dependent properties of enclosure walls from 45 examples of vitrified walls from Iron Age sites in Europe. For method details associated with the techniques used, please refer to the Methods section.

## Results: The Evolving Properties of Vitrified Enclosure Walls

**Figure 1:**
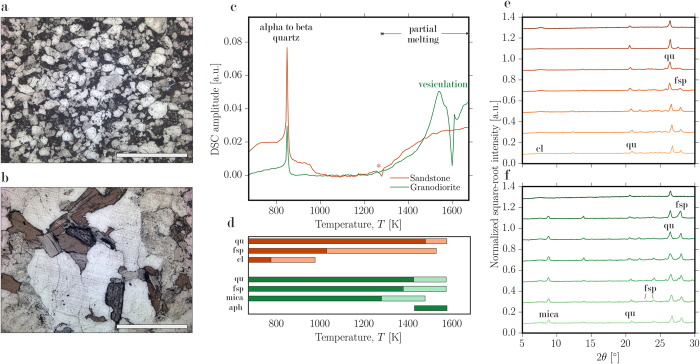
*In situ* thermodynamic and mineralogical constraint of the vitrification process in the end member lithologies granodiorite and sandstone up to 1500–1700 K. (**a**–**b**) Thin section photomicrographs of the (**a**) sandstone and (**b**) granodiorite sample microstructure prior to experiments for which the scale bar is 1.5 mm. (**c**) Differential scanning calorimetry shows events: the *α*−*β* quartz transition, the material solidus (coincident with a phase transition ‘*’ for the sandstone), and the onset of vesiculation in the granite. (**d**) Phase stability from *in situ* X-ray diffraction experiments where qu-quartz, fsp-feldsar, cl-clay and aphamorphous content, and the pale colours show that the phase is not stable. (**e**–**f**) XRD patterns collected *in situ*. Patterns from bottom to top represent 373 K to 1573 K at 200 K intervals for (**e**) the sandstone and (**f**) the granodiorite.

In Fig. 1 we show the evolution of the experimental materials as they are heated to temperatures anticipated for the generation of vitrified textures found in forts^13^,^14^. Using differential scanning calorimetry, we show that bulk melting temperatures may vary significantly from one lithology to the next (1293–1350 K – Lanhélin granodiorite and 1200–1340 K – Darley Dale sandstone determined by the onset of the partial melting endotherm; Fig. 1c). Using *in situ* high temperature X-ray diffraction, we are able to observe that this is a reflection of variability in starting mineralogy and its evolution during heating. During heating, feldspar, clay (in the sandstone case) and mica (in the granodiorite case) phases are depleted by 1400 K, yielding a quartz + liquid assemblage (Fig. 1d–f). On cooling, the liquid phase yields a glass which may exhibit partial devitrification textures involving quench crystals.

**Figure 2:**
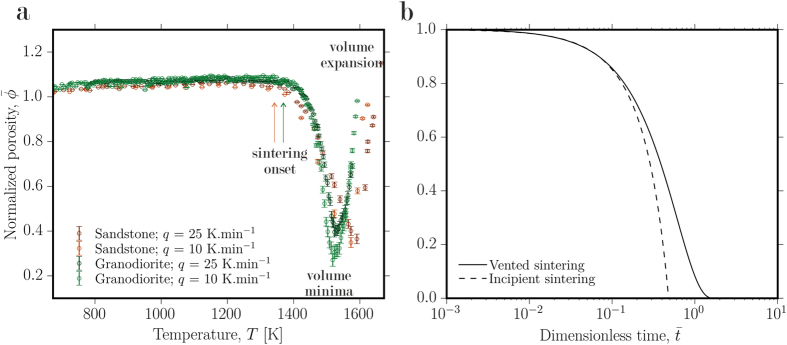
Sintering of particle aggregates. (**a**) Measurements of porosity changes in packs of sandstone and granodiorite particles showing (1) sintering begins in proximity to the solidus, (2) volume minima are reached when the sintering pack isolates pores and can no longer densify, (3) volume expansion ensues on continued heating and expansion of the newly isolated pore space. (**b**) The results of sintering kinetic theory for the vented sintering model^18^,^26^ and the incipient sintering model^19^, both cast in dimensionless form demonstrating that completion of sintering scales with the sintering timescale *λ* when the particles are viscous (above the solidus).

The interstices of the largest blocks in Iron Age walls contain material which is more fine-grained^13^. We observe that at temperatures near and above the solidus such packed grains densify at the expense of the bulk material porosity, both at relatively low and at high, rates of heating during particle-particle sintering^18^ (Fig. 2a). As the particles begin to melt, they act as partially viscous droplets containing remnant suspended crystals and undergo sticking and coalescence at particle-particle contacts^18^,^19^. These processes progress until a volume minimum is obtained where the porosity becomes isolated, and after which continued heating results in expansion of the now-isolated bubbles (see 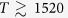
K in Fig. 2a). Both the onset temperature of sintering densification (coincident with the onset of partial melting at the solidus) and the decrease of the porosity upon continued heating are shifted in temperature and time due to 1) compositional differences of the rocks tested and 2) resultant differences in the temperature-dependence of the viscosity of the molten droplets produced. Our analysis of sintering behaviour underscores the significance of the behavioural differences during heating inherent in the widely varying lithology of the local rock. As the particles partially melt, the liquid viscosity evolves in time, strongly influencing the sintering rate. Although the effect of evolving partial melt composition is not explicitly measured here, we can scale the influence of this effect on the timescales of porosity loss between particles in fort walls using models for sintering of viscous droplets.

Sintering models are independent of gravitational effects when the Eötvös number Eo is less than unity, (i.e., where surface tension is the dominant driving force for porosity changes), and when the system compaction length is larger than the wall. These two cases are assessed here in turn. Eo is dependent on the liquid density *ρ*, the gravitational acceleration *g*, the particle length *R*, and surface tension *Γ* by 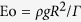. In an Iron Age wall, Eo < 1 when *R* < 3.5 mm, if we assign *Γ* = 0.3 for most dry silicate liquids^20^ and *ρ* = 2500 kg.m^−3^ for most silicate wall-forming compositions^13^. In Iron Age walls, this particle size limit is reasonable as particles in this range are typically the sizes that vitrify most readily due to rapid heat transfer^13^. The compaction length is 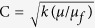 where *k* is the gas permeability between the sintering particles, and *μ*_*f*_ is the viscosity of the pore fluid (air in this case; *μ*_*f*_ = 10^−4^ Pa.s). If we assume 10^−12^ < *k* < 10^−11^ m^2^ for the granular material infilling the spaces between the large boulders by using a model for the permeability of granular media^21^,^22^, we obtain 22.4 < C < 707 m in the region of the solidus (by using 10^10^ < *μ* < 10^12^ Pa.s typical of high-silica partial melts at high temperature^23^,^24^). We can see that this is large compared with the wall heights on the order of 1–10 m^4^,^7^,^25^. From these considerations, we consider gravitational contributions to the sintering to be negligible and thus models for surface-tension-driven sintering sufficient.

Where gravitational compaction can be neglected, the sintering process is solely dependent on the initial particle packing porosity *ϕ*_*i*_ and the sintering timescale *λ* = *μR*/*Γ* where *μ* is the liquid viscosity or the viscosity of the partially molten suspension, *R* is the local radius of curvature of the particles or inter-particle pore space and *Γ* is the interfacial tension between the liquid and the fluid in the pore space (here, air)^18^,^19^,^26^,^27^. In Iron Age vitrified walls, *R* is highly polydisperse^13^,^28^ and, upon melting, *μ* will be a function of the evolving temperature and composition of the liquid. The Frenkel^19^ model for incipient particle-particle sintering and the Mackenzie & Shuttleworth^26^ model for continuum viscous sintering scale up the kinetics of bulk densification as a decrease in the normalized porosity 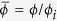 (where *ϕ* is the porosity) with normalized time 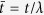 and demonstrate that it is *λ* and *ϕ*_*i*_ which exert the dominant control on the time required to sinter the stone walls. We present the dimensionless numerical solutions to these models to show how both are well scaled by *ϕ*_*i*_, rendering them applicable to different degrees of packing of particles (see full models in the Methods; Fig. 2b) and result in non-linear decays of porosity over times scalable only by *λ*.

**Figure 3:**
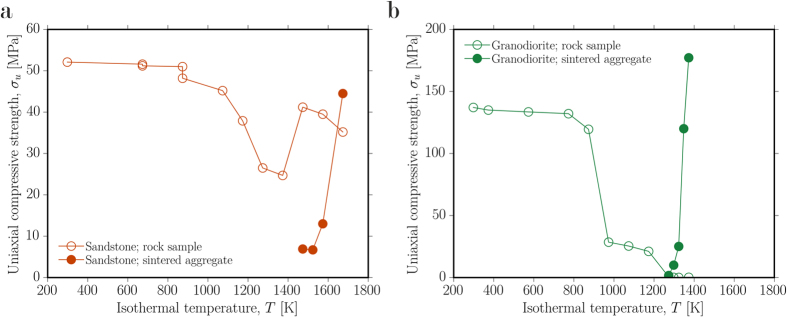
The strength of sintered packed-particle aggregates (sintered aggregate) compared with that of intact blocks of fort wall material (rock sample) for (**a**) the sandstone and (**b**) the granodiorite. Samples were heated for 5 hours isothermally at the temperature indicated and the strength was tested at room temperature on the experimental products.

The decrease of porosity by viscous sintering exerts a first-order effect on the material bulk strength^27^,^29^,^30^. Here, the strengthening effect was directly measured using a uniaxial strength metric to track the time- and temperature-dependent changes in material stiffness and load-bearing capacity for the two analogue compositions considered here (Fig. 3). We observe that [1] blocks of the material do become moderately weaker when initially exposed to high temperatures due to the proliferation of microcracks resulting from thermally-induced stress and, later, from vesiculation in the partial melt, in the case of the granite, and [2] a granular packing of the same material (analogous to the interstitial rubble between the dimension stones) becomes significantly stronger when exposed to the same conditions for a sufficient period of time. Here we use a 5-hour sintering period, shown to be a minimum for events in vitrified fort walls^13^. We conclude that for the initially granular materials, the kinetics of the strength increase are dependent on *λ*^27^. In a non-vitrified wall, the slip planes during destabilizing failure of the wall are along frictional contacts between blocks, and therefore, sintering of these contacts proportional in time to *λ*, results in stiffening of the bulk wall by an apparent mortaring effect. The complex case where the large blocks in the wall become weaker, while the fine-grained material strengthens, requires large-scale experimentation to determine the overall effect. However, these results demonstrate that to understand variability in wall strength from site to site, we need to understand how lithological variations influence *λ*.

### The Influence of Local Geology on Wall Properties During Vitrification

The parameters in the sintering timescale that vary most significantly between Iron Age fort lithologies are [1] the material solidus temperature and [2] the temperature dependence of the evolving viscosity *μ* during partial melting. To explore the first-order variability in *μ*, we have employed both bulk- and glass-compositional data from the walls of 45 Iron Age forts across Europe (including experimentally produced glass from partial-melts of protoliths)^1^,^3^,^4^,^12^,^14^ and reference material compositions of Moine assemblages from Scotland^31^ together with a multicomponent silicate liquid viscosity model^23^ in order to obtain the composition-dependence of the fragility *m* of the liquid viscosity. The fragility is a measure of the temperature-dependence of the viscosity in the high viscosity region (close to the glass transition of the liquid intersected at a cooling rate of 5 K.min^−1^) and is given by^23^


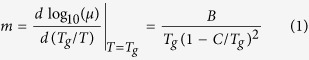


where *T* is the temperature, *B* and *C* are coefficients given by the multicomponent model for silicates^23^ and *T*_*g*_ is the glass transition temperature (the temperature of glass formation on cooling). In this case, *T*_*g*_ represents the temperature below which further sintering of the stonework cannot occur as well as serving as a reference temperature for assessing *m*. High values of *m* indicate that the liquid viscosity exhibits a strong temperature-dependence, such that the viscosity will drop quickly as the temperature continues to increase.

**Figure 4:**
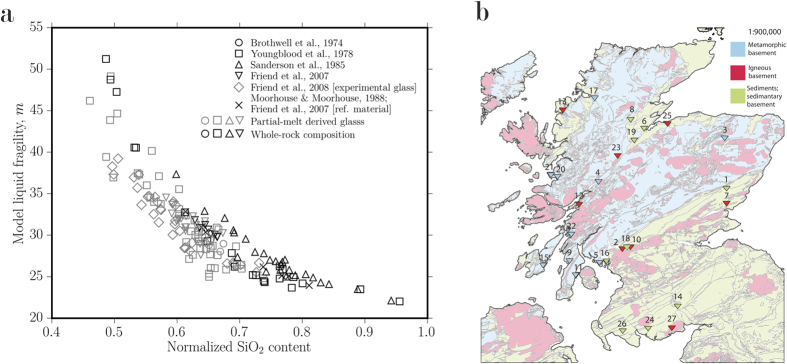
(**a**) The liquid fragility *m* for 44 vitrified fort compositions^1^,^3^,^4^,^12^,^14^ or the partial melts thereof constructed in the Iron Age across Europe and Scottish Moine assemblage reference samples^31^ as a function of the silica content (when normalized by the total measured element abundance). (**b**) A map of Scottish fort locations with corresponding simplified bedrock geology. While forts exist throughout Europe, Scottish forts are the best documented to date. Fort numbering: 1- Finavon, 2- Craig Marloch Wood, 3- Tap O’North, 4- Dun Deardail, 5- Dunagoil, 6- Craig Phaidrig, 7- Laws of Monifieth, 8- Knockfarrell, 9- Dunskeig, 10- Dumbarton Rock, 11- Carradale, 12- Dun MacUisnichan, 13- Art Dun, 14- Mullach, 15- Trudernish Point, 16- Cumbrae, 17- Dun Lagaidh, 18- Sheep Hill, 19- Urquhart Castle, 20- Eilan-nan-Gobhar, 21- Eilan nan Ghoil, 22- Duntroon, 23- Torr Duin, 24- Trusty’s Hill, 25- Doon of May, 26- Castle Finlay, 27- Mote of Mark. This map was produced using QGIS Vienna 2.8.2 (http://www.qgis.org/en/site/) and the British Geological Survey’s open-access DiGMapGB-625 (http://www.bgs.ac.uk/products/digitalmaps/digmapgb_625.html) for the basement geology and the positions of the vitrified forts provided by Sanderson *et al*.^3^ (vitrification evidence at these locations is variable). Reproduced with the permission of the British Geological Survey ©NERC. All rights reserved.

Use of the whole-rock compositional data simulates the scenario where the entire rock is molten, whereas use of the glass compositional data corresponds to the scenario where partial melts are generated from *in situ* melting of the walls (Fig. 4; where SiO_2_ content is normalized by the totals). We observe large differences in the computed values of the fragility *m*, across the range of fort wall compositions. This is a clear indication that fort wall lithology determines the viscosity during subsequent non-isothermal high-temperature firing. Once known, *μ* can be used to obtain *λ*, which tracks the time required for densification of the wall. Here we emphasize that large blocks will take much longer to reach thermal equilibrium than small blocks, which confirms why small particles mortaring the interstices of larger blocks are often the most vitrified whereas large blocks often remain relatively unaltered by the high temperature events (as noted for the Wincobank site in Sheffield, U.K.^13^).

In 1938, Childe & Thorneycroft^28^ constructed a stone-built wall in order to test the feasibility of vitrification *in situ*. They used inch-sized basaltic rubble interstitial to fire-clay bricks and considered their conclusions generally relevant to Iron Age vitrified walls. However, in doing so they neglected both the material and the lengthscale considerations that we have explored above. Partially on the basis of the Childe & Thorneycroft^28^ reconstruction results, Mackie^7^ rejected a strengthening effect as a relevant consideration for interpreting vitrification. Above we have demonstrated that strengthening is a likely consequence of intense and thorough vitrification processes that result from heat above the material solidus. Below we assess the degree to which these findings might elucidate the motives of Iron Age fort builders.

## Discussion of Vitrification in Iron Age Scotland

There is a strongly kinetically-limited portion of wall vitrification processes which can be captured by the sintering timescale *λ* and the material solidus that approximately scale the vitrification intensity and strengthening potential. At times less than *λ*, sintering has not proceeded and strengthening may not be expected whereas at times greater than *λ*, sintering is complete and the strength of the particles between large blocks has increased significantly (Fig. 3). The long duration at high temperature to which these walls were exposed^13^,^14^ is especially conducive to pervasive sintering of the small particles, leading to maximum strength increases in the areas interstitial to large blocks. It is the lithology from which forts are constructed that most strongly controls the temperature dependence of the liquid viscosity (captured by the liquid fragility *m*), and therefore the sintering efficiency and the strengthening potential. For example, if fort firing times and temperature distributions were similar from site to site, attributable to similar constructions and similar fuels for the fires, then settlements in the vicinity of surface-exposed granite plutons, such as the Mote of Mark fort, Scotland, are more likely to exhibit a greater vitrification than, for example, the Wincobank hill fort built from sandstone (Fig. 3b), due to their differential behaviour during fort firing. In Fig. 4 we show the distribution of enclosures in Scotland for which partial or thorough vitrification has been reported along with a simplified basement geology which approximates the local lithologies available to the fort builders.

Vitrification as a prehistoric cultural practice is still largely enigmatic and many factors must be taken into account. Conclusions based solely on experimental work are fragile at best. In the absence of more direct evidence, it is not yet clear that the material properties and the strengthening effect were of interest to the builders of Iron Age enclosure walls. Not all forts are vitrified around their entire perimeter^12^, suggesting that either vitrification was unsuccessful or that thorough partial melting was not the desired result and there is evidence that firing occurred after initial construction^8^. Moreover, the association of Iron Age enclosures with fortification or defence has been challenged^9^,^32^,^33^. Therefore, the strengthening effect may have been incidental. Ralston^2^ summarizes by stating that in well-excavated examples^7^ vitrification is most prominent local to timber lacing used to construct the wall itself, presumably where the temperatures were highest. He concludes that thoroughly vitrified examples are likely to have been formed during the systematic destruction of the site after capture by successful attackers, consistent with observations that vitrification products include fused items from the period of occupancy^7^. However, this view is in contrast with Youngblood *et al*.^12^ who propose that timber-lacing internal to the wall construction would be insufficient to sustain fires at the required temperatures for the required times and that the fuel cost would be unreasonably large^1^, and instead suggest that the walls were perhaps covered in peat to produce long-lasting, reducing, high temperature conditions.

Two possibilities appear to us to be most tenable: [1] fort vitrification was intentional and constructional and the purpose was to build strong walls^29^, or [2] that this practice was intentional but destructional and that the details of vitrification and the resultant strength increase were incidental results. While the latter has become a leading theory, the former has been dismissed primarily on the basis of failed large-scale reconstruction experiments. We propose that if the former is indeed a possibility, then the pyrotechnology of Iron Age engineers was not ubiquitously successful as an engineering solution in sites where the building materials were not conducive to melting and sintering. Additionally, other technologies for strengthening walls may have replaced vitrification if wall-firing was repeatedly shown to be less successful in regions of refractory geology. Were the Iron Age wall builders in fact interested in strengthening their walls, it is likely that they would have experimented with different techniques for doing so and firing of the wall possibly represents one such experimental solution that was successful in regions of predominantly igneous or metamorphic basement in Scotland (Fig. 4), but failed to be readily exportable into Europe where available building materials were not as effectively melted. Whatever the motive for firing walls, the local geology from which Iron Age forts are built is a key factor in explaining the variability of vitrification from site to site across Europe.

## Methods

High temperature X-ray diffraction was performed at the Natural History Museum, London, using a Nonius X-ray diffraction instrument equipped with an INEL curved 120° 2θ position-sensitive detector (PSD) with the beam and sample in static reflection geometry. The system consists of a GeniX Cu high-flux X-ray system equipped with a Xenocs FOX two-dimensional 10_30 P mirror to generate high-brightness Cu K*α* radiation. Samples were heated *in situ* using an Anton Paar heating stage (HTK-10) with a platinum strip from room temperature to 1573 K with a stepwise heating profile at an average rate of 10 K min^−1^. The high-brightness X-ray source allowed rapid data collection of isothermal diffraction patterns, each of only 2 min duration, at 50 K intervals. Thus, a sequential 0–120° 2θ snap-shot visualization of the changes in mineralogy throughout the heating cycle was recorded by the PSD. Minerals were identified using the PDF-2 database cards from the ICDD (International Centre for Diffraction Data, http://www.icdd.com). The temperature at which primary mineral peaks began decreasing in intensity marked the onset of phase instability and the temperature at which these peaks became indistinguishable from background defined the phase-out point.

Differential scanning calorimetry was performed using a Netzsch Pegasus 404 simultaneous thermal analyser. 30–70 mg of rock material, gently hand-crushed in an agate mortar, was loaded into lidded platinum crucibles. The experiments were performed in a 20 ml.min^−1^ argon flow. The mass and unconverted DSC signal (μV.mg^−1^) were measured continuously at high resolution 200 pts.K^−1^. Baseline empty-crucible runs were subtracted from the resultant data. The convention *exothermic down* is adopted herein.

Optical dilatometry was performed using gently hand-crushed material sieved to <125 μm particle sizes. The particles were packed into 3 mm diameter free-standing cylinders which were loaded into a Hesse Instruments camera-furnace array. 2D images were captured continuously during heating at 10 or 25 K.min^−1^. Conversion of 2D sample cross-sectional images to the 3D porosity metric was achieved by numerically integrating the sample edge position about the central axis of symmetry. This is done by defining the distance of the edge from the central symmetry-axis as *β*, the sample height as *L* and the vertical direction from base to top as *y*, so that the integral for sample volume is


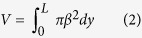


The volumes in voxels were converted to porosity *ϕ* by knowing an initial porosity *ϕ*_*i*_. The initial porosity was determined by the volume, mass and powder-density where the powder-density was measured in a helium pycnometer. Then *ϕ* = 1 − *V*_*i*_(1 − *ϕ*_*i)*_)/*V* where *V*_*i*_ is the initial sample volume.

The sintering theory shown in Fig. 2 can be summarized by presenting two non-dimensional models. First, the timescale *λ* includes a lengthscale *R*. If we initially state that this is the radius of the particles *R*_*p*_ so that the timescale becomes *λ*_*p*_, we then have a dimensionless time 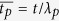. This allows us to use the dimensionless Frenkel^19^ model for incipient sintering between particles where the porosity decreases due to the growth of inter-particle necks. This model is


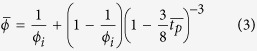


If, however, we assume that the lengthscale in the timescale *λ* is the inter-particle pore radius *R*_*a*_ then we have the dimensionless time 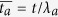 and the Mackenzie & Shuttleworth^26^ model for an array of shrinking pores as follows (see ref. 15)


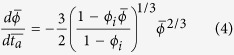


Laboratory deformation experiments were performed to test the material strength evolution. To do this, 20 mm-diameter cylindrical cores were drilled from single blocks of Darley Dale sandstone and Lanhélin granodiorite and precision-ground to a nominal height of 40 mm. These samples were heated at 10 K.min^−1^ in a box furnace to temperatures 400–1800 K and held isothermally for 5 hours. 5 hours represents the time required to ensure thorough thermal equilibrium in these materials^29^. Hand-crushed powders of the same materials were loosely packed in alumina tubes and heated under the same conditions before being drilled and ground to the same dimensions. Helium pycnometry was used to determine the connected porosity of each sample prior to and following exposure to high temperature. Finally, the strength metric chosen is the uniaxial compressive strength (UCS). For our UCS tests, the 20 mm-diameter cylindrical samples (each symbol on Fig. 3 represents a unique experiment; we therefore performed 12 and 11 tests on thermally stressed samples of Darley Dale sandstone and Lanhélin granite, respectively, and four and five tests on sintered aggregate samples of Darley Dale sandstone and Lanhélin granite, respectively) were deformed at a constant axial strain rate of 10^−5^ s^−1^ until macroscopic failure. The samples deformed for this study respected the convention that the grain/crystal size to sample diameter ratio should be at least 10:1 (Fig. 1a,b). Following the strength tests, the remnant material (broken fragments) was crushed to the pore-free particle sizes; the density of this powder was then determined by helium pycnometry, which allowed post-experimental computation of the total porosities of all of the experimental samples. All samples were vacuum-dried for 48 hours at 313 K prior to experiments and a lubricating wax was applied to both ends of the samples to limit stress due to friction between the pistons and the sample.

## Additional Information

**How to cite this article:** Wadsworth, F. B. *et al*. Local geology controlled the feasibility of vitrifying Iron Age buildings. *Sci. Rep.* **7**, 40028; doi: 10.1038/srep40028 (2017).

**Publisher's note:** Springer Nature remains neutral with regard to jurisdictional claims in published maps and institutional affiliations.
